# Understanding older women’s decision making and coping in the context of breast cancer treatment

**DOI:** 10.1186/s12911-015-0167-1

**Published:** 2015-06-10

**Authors:** Kate J Lifford, Jana Witt, Maria Burton, Karen Collins, Lisa Caldon, Adrian Edwards, Malcolm Reed, Lynda Wyld, Kate Brain

**Affiliations:** Cochrane Institute of Primary Care and Public Health, School of Medicine, Cardiff University, Neuadd Meirionnydd, Heath Park, Cardiff, CF14 4YS UK; Institute of Palliative Care, Policy and Rehabilitation, Kings College London, Cicely Saunders Institute, Bessemer Road, Denmark Hill, London, SE5 9PJ UK; Centre for Health and Social Care Research, Sheffield Hallam University, 32 Collegiate Crescent, Sheffield, S10 2BP UK; Academic Unit of Surgical Oncology, University of Sheffield Medical School, Royal Hallamshire Hospital, Glossop Road, Sheffield, S10 2RX UK; Brighton and Sussex Medical School, University of Sussex, Falmer, Brighton, BN1 9PX UK

**Keywords:** Breast cancer treatment, Old age, Decision making, Coping, Deliberation

## Abstract

**Background:**

Primary endocrine therapy (PET) is a recognised alternative to surgery followed by endocrine therapy for a subset of older, frailer women with breast cancer. Choice of treatment is preference-sensitive and may require decision support. Older patients are often conceptualised as passive decision-makers. The present study used the Coping in Deliberation (CODE) framework to gain insight into decision making and coping processes in a group of older women who have faced breast cancer treatment decisions, and to inform the development of a decision support intervention (DSI).

**Methods:**

Semi-structured interviews were carried out with older women who had been offered a choice of PET or surgery from five UK hospital clinics. Women’s information and support needs, their breast cancer diagnosis and treatment decisions were explored. A secondary analysis of these interviews was conducted using the CODE framework to examine women’s appraisals of health threat and coping throughout the deliberation process.

**Results:**

Interviews with 35 women aged 75-98 years were analysed. Appraisals of breast cancer and treatment options were sometimes only partial, with most women forming a preference for treatment relatively quickly. However, a number of considerations which women made throughout the deliberation process were identified, including: past experiences of cancer and its treatment; scope for choice; risks, benefits and consequences of treatment; instincts about treatment choice; and healthcare professionals’ recommendations. Women also described various strategies to cope with breast cancer and their treatment decisions. These included seeking information, obtaining practical and emotional support from healthcare professionals, friends and relatives, and relying on personal faith. Based on these findings, key questions were identified that women may ask during deliberation.

**Conclusions:**

Many older women with breast cancer may be considered involved rather than passive decision-makers, and may benefit from DSIs designed to support decision making and coping within and beyond the clinic setting.

## Background

The extent to which older women with breast cancer are involved with and cope with treatment choices, and the potential need or value of decision support interventions, is not well described. Primary endocrine therapy (PET; hormonal therapy) can be just as effective as surgery followed by endocrine therapy for a subset of older women (75 years and over) with oestrogen receptor positive breast cancer and limited life expectancy. It is therefore a good alternative for this group as survival is equal for the two treatments, although local control is inferior with PET [1]. The decision as to which treatment is best for these women is preference-sensitive, highlighting the need for appropriate decision support for older women with breast cancer. The decision involves making trade-offs, for example women may have to consider pain and potential morbidity associated with surgery against potentially having a residual palpable breast lump which may serve as a reminder of their cancer if they choose PET. Having PET also involves more frequent attendance at follow-up appointments over a longer time period, and there is the risk that it may stop working after a couple of years. Should the cancer escape control with PET, a change of management is necessary. This can be surgery or alternative lines of PET. However, alternative PET options tend to be effective for shorter periods and these options are eventually exhausted. In effect, older women are being asked to gamble whether they will die of something else before the breast cancer escapes control by the PET and whether they are prepared to risk this to avoid surgery. It is therefore a complex and potentially difficult decision.

Previous studies of treatment decision making in this patient group suggest that most women do not actively ask questions of healthcare professionals, but rather look to them for guidance [2]. Older women’s main concerns related to non-medical aspects such as maintaining their independence, which they did not discuss with their healthcare professionals. Similarly, Burton and colleagues [3] found that, while older women appreciated being offered a choice of PET or surgery, many sought guidance from their healthcare professionals to recommend or approve a treatment choice. Women often chose their treatment based on past experiences and pre-existing knowledge, and usually with the goal of either removing the cancer or avoiding surgery [3]. These studies highlight the need to engage older women with breast cancer in discussions with their healthcare professionals about their treatment.

Decision support interventions (DSIs) have been shown to improve patient knowledge and involvement in decision making [4]. When developing a complex intervention, such as a DSI, a theoretical underpinning should be used to guide the content [5]. The Coping in Deliberation (CODE) framework [6] is suited to understanding treatment decision making as it includes emotions and coping as well as cognitive processes. The framework integrates cognitive and emotional appraisals of the health threat and decision (primary appraisals) and potential coping strategies which are identified (secondary appraisals) throughout the deliberation process. These concepts are based on Lazarus and Folkman’s [7] transactional theory of stress, appraisal and coping, and Leventhal’s [8] self-regulatory model of illness perceptions [6]. Deliberation is a process comprised of a number of stages throughout which information is considered, appraisals made (i.e. that there is a choice, what the options are, the pros and cons of each option), preferences formed, and finally a decision made [9]. Appraisals at each stage of the deliberation process can influence other stages (both previous and subsequent) of the deliberation process and ultimately influence the treatment decision. The CODE framework has previously been adapted to understand women’s decisions about risk reducing bilateral salpingo-oophorectomy and to form the basis of a DSI [10, 11]. Examining coping and decision making processes is particularly important when DSIs are designed for a specific age group, because the use of different coping strategies across the life span [12] may influence not only the content of the DSI, but also its use and implementation. There is evidence to suggest that older patients tend to prefer less participation in treatment decision making, previously described as a ‘passive’ approach [13]. Older people may in fact be more likely to use efficient coping strategies which conserve processing resources, as well as strategies which have been effective in the past [12]. Older people have also been shown to use positive re-appraisal and advanced coping strategies such as decentring and focusing on long-term, rather than short-term, goals [12]. Their appraisal of stress, and consequent need for coping strategies, is therefore potentially different to those of younger people [12].

The present study is part of the Bridging the Age Gap in Breast Cancer programme which aims to provide evidence-based guidance for the treatment of older women with oestrogen receptor positive breast cancer in the UK, and to minimise unwarranted variation in the management of this patient group. The analysis in the study by Burton and colleagues [3] focused on women’s informational needs and preferences for the decision between surgery and PET. The present study was a secondary analysis of the data to examine women’s cognitive and emotional representations of the treatment decision, as well as coping resources. The CODE framework was used to understand older women’s coping and decision making processes in order to guide the content of DSIs designed to engage them in breast cancer treatment decisions.

## Methods

### Sample

National Research Ethics approval was obtained (12/LO/1722) and Research Governance approval for each participating centre. Patients were recruited from five Breast Units, over three regions across the UK; Yorkshire and Humber (Doncaster and Sheffield), East Midlands (Derby and Leicester) and South Wales (Cardiff). Women aged 75 years and older who had been diagnosed with oestrogen receptor positive breast cancer (in the last 5 years) and had been offered a choice of PET or surgery with endocrine therapy, were invited to take part in the study (see [3] for more details).

### Procedure

Following written informed consent, interviews were conducted by experienced qualitative researchers (MB and KL) predominantly in the participants’ own homes. Interviews were audio-recorded and transcribed verbatim. The interviews were designed to understand women’s treatment decision making, information needs and preferred media for information [3]. The semi-structured interview schedule (see [3] supporting information) included the following topics: breast cancer diagnosis, perceptions of treatment, treatment decision making, sources of information and preferred formats of DSIs. The schedule was not based specifically on the CODE framework; however, areas relating to elements of Leventhal’s [8] self-regulatory model of illness perceptions (one of the theoretical underpinnings of the CODE framework) were included, as well as aspects of the decision making process (e.g. which factors influenced their decision).

### Analysis

**Fig. 1:**
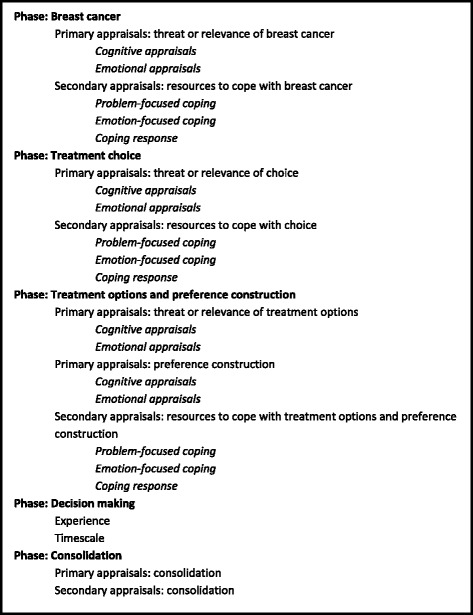
Coping in Deliberation analysis coding framework

The interview data generated multiple analyses. The primary analysis focused on the information needs and preferences for decision making and used an inductive approach and is reported elsewhere [3]. For the present study, a secondary analysis of the interviews was conducted by imposing a conceptual framework [6] to identify specific decision making appraisals and coping strategies. The present report therefore summarises results from an additional set of analyses from the same set of interviews. After familiarisation with the data, transcripts were coded (by JW) according to the CODE framework phases (Fig. 1, [6]). Twenty percent of the coded transcripts were reviewed (by KL and KB) and discrepancies were resolved through discussion. Reviewing 20 % of the coded transcripts was a pragmatic decision. Quotes were then charted within the relevant code(s). The data within the chart were then reviewed and used to adapt the questions in the generic CODE framework (Tables 1, 2, 3, column A-Generic CODE question) to the PET vs. surgery with endocrine therapy decision (see [11] for further details). Quotes within the tables are good examples of the issue being raised that relates to the relevant CODE question, but they are not necessarily representative of the sample in terms of their specific content. Because the analysis was secondary, data that were deemed not relevant to the decision making process and coping (such as details of the diagnosis and preferred media for information) were not included.

**Table 1:** Appraisals of breast cancer

	A-Generic CODE question	B-Adapted CODE question^	C-Example quote†
**A. Primary appraisals: breast cancer**	What are the causes?	What caused my BC?	*“I was shocked because it’s not in* [my] *family … but my husband was a heavy smoker, now I don’t know whether that was anything to do with it.”*
	What does this mean?	**What is the prognosis for BC?**	*“Oh crikey, this is end of me life. This is end of me.”*
	How long will this last?	**What are my chances of surviving this?**	*“…I don’t know exactly the numbers but it’s pretty high the recovery rate.”*
	How relevant/threatening is this?	**How will BC affect my life?**	*“Oh no, urgh, might be bald for the wedding”*
		How important/ threatening is BC?	*“I didn’t realise it was as serious until* [name] *said to me, ‘It’s quite serious you know?’ And I said, ‘Oh is it?’”*
		**What experiences do I have of cancer?**	*“…my mother-in-law had breast cancer and she had it removed and she waited until the lump was that big. …I was with her actually but she didn’t last very long.”*
	How do I feel about this?	**How do I feel about having BC?**	*“I mean when you’re older it’s, it doesn’t matter really as much at all. … I mean it’s still a bit scary but you know we’ve all got to get something…”*
		How do I feel about the way my BC was diagnosed?	*“I was shocked, I know I was shocked at first because I mean I’d examined my breasts regularly and I had no idea and so I was shocked…”*
		How do I feel about the potential symptoms of BC I could experience?	*“I only was worrying about the pain I’m going to get in time…”*
		How do I feel about the impact BC could have on my life?	*“Well I suppose it's put a cloud over life a bit.”*
B. Secondary appraisals: resources to cope with breast cancer*	Can I find out more?	Can I find out more about breast cancer from books/the internet?	*“…I read it so I can hold in my memory or look forward to probably* [it's] *going to tell me* *what to expect … So I read every little paper I go (anywhere) and see about cancer…”*
	Can my physician help?	Can my doctor/nurse give me more information?	*“…I was shocked* [at breast cancer diagnosis] *… then of course I wanted to know what was going to happen …everything was explained to me and as I said this nurse I asked her questions and it was ok after that. I didn’t worry.”*
	Can I talk to my family/friends?	Can a friend/relative help me find more information?	*“…fortunately my daughter will come from* [name] *and, you know, look after me, and her husband being a doctor, she sort of knows what questions, what I ought to be asking…”*
		Can I get some instrumental support from family/ friends?	*“…one of my daughter-in-law’s sister … bought me a stack of vitamins and - and so I still take those actually.”*
		Can I get emotional support from family/friends?	*“…we went, my daughter-in-law went back with me and he said, ‘We’re very sorry; it was cancer.’”*
	Can I change how I feel about this?	Can I see a positive side to having BC?	*“I’ve always said people are a lot worse and when I got to my age and I got this I thought well I’m on me way… I don’t worry about dying…”*
		Can I accept my BC?	*“I’ve always said people are a lot worse and when I got to my age and I got this I thought well I’m on me way… I don’t worry about dying…”*
		Can I find a way to relax?	*“…you need to, to try and accept the cancer as well and things like relaxation, reflexology and reiki…”*
	Could I ignore this information?	Can I distract myself/ block thoughts about BC?	*“…I think then I just shut my mind to the fact that I had even cancer…”*
	Can I find strength in my faith?	Can I find strength in my faith?	*“…I have faith. We both have, belong to the church and I’ve clung through all these things I’ve clung onto my faith even by my finger ends sometimes…”*

^Those in bold are questions that were explored by many women during primary appraisal, the other questions were explored by fewer women

†Good example quote of the issue being raised but the specific content is not necessarily representative of the sample

*Can I express my fear?” was not adapted

BC = breast cancer

**Table 2:** Appraisals of treatment choice

	A-Generic CODE question	B-Adapted CODE question^	C-Example quote†
**A. Primary appraisals: treatment choice**	Why is there a choice?	**Is there a choice in my case?**	*“…they weren’t going to operate because of my frailty and I thought that was probably a way of saying I was too old.”*
	What does this choice mean?	**What is the scope of the choice I have?**	*“…he did give me the option of what he could do, he could just take the lump out, he could remove the breast, there’s something else.”* Interviewer: Hormone treatment? *“I don’t remember that but I know he said if I had just the lump taken, oh and he could also rebuild it at the same time if I wished and all this you know, take the lump out…”*
	How long do I have to decide?	What is the timeline for making this decision?	*“…you see you don’t have time, because you want it done there and then. So you don’t… you don’t have a lot of time to think about it.”*
	Who can decide?	Who is responsible for making this decision?	*“…*[my son] *did say to* [the doctor] *‘if it was your wife what would you recommend her to do?’ and he said ‘I can’t answer that’ he says, you know, ‘it’s your mother’s decision. She has to decide for herself.’”*
	How do I feel about choosing?	How do I feel about the fact that there is a choice?	*“I was quite happy, quite happy with the choice that I was given.”*
		(How do I feel about the fact that there is no choice for me?)	*“I wasn’t given a choice no.”*… Interviewer: … would you have liked a choice? *“I think I would really. I don’t know what I would of chosen though thinking about it.”*
		How do I feel about taking part in making a decision?	*“It would have been nice to have been offered the alternative which me pushing it”*
B. Secondary appraisals: resources to cope with choice*	What support do I have?	Can my doctor/ nurse help me make this decision?	Interviewer: … how much do you think the consultant played a part in your decision? I mean I suppose…? *“I think she played a big part, because I think she put it so clearly that it was easy to make a decision.”*
		Could my family / friends help me make this decision?	*“…my daughter, (…) she is a qualified nurse …she just sort of said ‘ask them this and ask them that’”*
	Can I deal with choosing myself?	Can I cope with making this decision all by myself?	*“my youngest daughter didn’t feel good about me not having the* … *treatment, whatever treatment they gave me you know away from having the tablets. My younger daughter she is still mad up to today.* …* but I say no it’s my choice.”*
	Can I transfer this decision?	Can my faith help me make this decision?	*“I feel if you pray, if you have a faith and you pray and then to put… you leave it in God’s hands.”*
	Can I transfer this decision?	Can I let someone else decide for me?	*“…I said to my son-in-law, ‘Honestly* [son-in-law’s name]*, give me your opinion. Now do I really need the operation, or…?’ I didn't say I would go with what he said, I said, ‘I want you to tell me which one to take, the operation or the medication?’.”*
	Could I refuse to choose or defer the decision?		

^Those in bold are questions that were explored by many women during primary appraisal, the other questions were explored by fewer women

†Good example quote of the issue being raised but the specific content is not necessarily representative of the sample

* "Can I distract myself?" was not adapted

BC = breast cancer

**Table 3:** Appraisals of treatment options and preference construction

	A-Generic CODE question	B-Adapted CODE question^	C-Example quote†
A. Primary appraisals: treatment options*	What are my options, what do they involve?	**What does mastectomy/lumpectomy/hormone therapy (HT) involve?**	*“…I think I’d like explained what would happen in the surgery and the after effects, in relation to what would happen if you don’t have it done and have the hormone treatment.”*
	What are the risks and benefits?	**What are the risks and benefits and prognosis of mastectomy/lumpectomy/HT?**	*“…I would want to know what the results was of the people that had an operation… surgery, or people that had had the tablets…”*
	What are the follow-on decisions?	**What are the consequences and follow-on decisions of mastectomy/lumpectomy/HT?**	*“I may have just had the lumpectomy if it had been easier to have the radiotherapy. That did have a bearing I must admit…”*
	How would this affect my life?	How would mastectomy/lumpectomy/HT affect/fit into my life?	*“…maybe I'm a bit wary if I had an operation would I then be, not be as active as I am?”*
		How would mastectomy/lumpectomy/HT affect/fit into important others’ life?	*“…you don’t really want to be bothering other people … I have two sons … they were supportive but you don’t want to have to have them trailing and interfering with their jobs …””*
	Do I have experiences that could help me imagine what it would be/feel like? What did others decide?	**What experiences do I have of surgery/mastectomy/lumpectomy/HT?**	*“…what I’ve heard about it, people and friends going, I would not want to be going every day for radiotherapy somewhere. I thought this I cannot do.”*
	How do I feel about this option?	**How do I feel about having surgery and about experiencing the potential consequences of surgery?**	*“I was frightened about the fact that I might have had to have an operation…”*
		How do I feel about radiotherapy (and chemotherapy)?	*“Radiotherapy, I’d got a dread of that…”*
		How do I feel about breast reconstruction?	*“…when I think about that* [reconstruction of breasts], *I think, you’re having something taken out and then you’re having something put back in! … it doesn’t sit right with me.”*
		How do I feel about taking tablets every day?	*“But er, no way did I want to take tablets…”*
B. Primary appraisals: preference construction**	Is this the right time?	What do I want to do at my age?	*“…I had no intention in being operated on … I had no intention. I was in my eighties, I thought if I got to pop off I’ll pop off quietly.”*
		Could I still change my mind/is this option reversible?	*…I thought, “No, I don’t, I don’t fancy having it* [the breast] *off.” … I’ll try, I’ll try, because I felt if it was to come off I could have it done later.*
	Is this option congruent with my/ my family’s/my doctor’s beliefs, goals and values?	**What is my doctor’s recommendation?**	*“Well they said that the operation wasn’t necessary, it wasn’t a choice duck so I thought well that’s grand, … I didn’t query it duck, because they’re professional people, I accepted it…”*
		**Is this option congruent with my personal goals, values and beliefs?**	*“…‘I am not having chemo, and I’m not having radium’, I said, ‘I don’t want to live any longer, but I do want to stay in my own house as long as I possibly can … what I insisted on was, trying to give me the best quality of life they could give me, not to live longer, to stay here.”*
		Is this option congruent with important others’ goals, values and beliefs?	*“My son was with me and he really agreed with me when I just wanted to have treatment straightaway I wanted surgery and I didn’t really want to delay it…”*
	n/a	**Do I have a gut feeling/reaction?**	*“…I’d already made my mind up because I knew that it was cancer before (I) – you know and in my own mind and I'd made my mind up that I was having … the breast taken off.”*
	n/a	Does one option feel better/safer than the others?	*“…I can’t explain it really, but I felt happy to go that way and to have the tablet.”*
C. Secondary appraisals: resources to cope with treatment options and preference construction***	Can I seek support/information elsewhere?	How can I find out more about my options?	*“…the only reason I knew I think was because my sister bought me a book and that told you a lot more…”*
	Who can I discuss this with?	Can I talk to someone who has had BC and BC treatment?	*“…she said come and see her you know so we both poled over and she bared all and she said she wouldn’t have a lumpectomy because of the radiotherapy which finished her off. So you know it was quite useful information.”*
	Are there any more alternatives or additional actions I could take?	Can I take action against my BC in another way?	*“…she’s been giving me supplements and put me, been put on a regime and things like that, which has helped me…”*
		Can my body cope with surgery/am I healthy enough?	*“…I explained to them I think, that it wasn’t that, it was just that I, I felt, I knew I couldn’t cope with that. And also, I just, I just couldn’t. It wasn’t anything to do with my body or anything like that, but I knew that I wasn’t, I wasn’t fit, to be quite honest…”*
	What support do I have?	What instrumental support do I have?	*“…you don’t really want to be bothering other people, I mean, my son lives nearby and he was very supportive. … so they were supportive but you don’t want to have to have them trailing and interfering with their jobs and what have you…”*
		What emotional support do I have?	*“Oh I talked to my family about it, yeah, and anybody who sort of, and my friend… I’ve got one very close friend and I always talk to her about everything…”*
	n/a	Can I cope emotionally with treatment/am I emotionally strong enough?	*“The only thing I was frightened of, and I was petrified, anaesthetic … And I spoke to an anaesthetist the day before and he were brilliant and he explained it all and he said, you will be fine.”*
	Can I find the answer in my faith?	Can I find strength in my faith?	Interviewer: …did you think ‘oh dear I need to have it off’ or did you think… *“no I didn’t actually. I just – I just thought it was another answered prayer. I did not question anything and I have never had any trouble with it.”*
	Can I make this decision easier?	Can I ignore information about options and make a decision without looking into details?	*“I’ve never gone into things to do with illness too deeply. I’ve always thought ignorance is bliss … Don't get me wrong, I’m sure it’s very good that the information is there, but it doesn’t, I don’t think it was my way of doing it, I prefer not to know about all the things that could go wrong…”*

^Those in bold are questions that were explored by many women during primary appraisal, the other questions were explored by fewer women

†Good example quote of the issue being raised but the specific content is not necessarily representative of the sample

* “What would happen if I wait?” was not adapted

** “Do I feel ready?” and “How likely is it that I will experience regret?” were not adapted

*** “Would my family/partner support me if I choose this option?”, “Is there anything I can do to change how I feel about this option?”, “Could I defer the choice?”, “Should I follow my gut feeling/ intuition?” and “Can I let someone else decide?” were not adapted

BC = breast cancer, HT = hormone therapy

## Results

### Sample characteristics

Interviews were completed with 36 women (we do not have accurate data on the number of women invited); however, one interview was excluded due to a recording failure. Thirty-five patients were therefore included, whose ages ranged from 75 to 98 years (median age 83 years). The majority of women were recruited from Yorkshire and The Humber (n = 27) with a further five from East Midlands and three from South Wales. Women were between 3 and 96 months post-diagnosis when interviewed. The majority of the data for calculating the time since diagnosis is based on women’s reports, some is based on clinician reports, and is therefore only approximate. Twenty three women had received PET (median age 85 years, range 76 to 98 years) and twelve had undergone surgery (median age 79.5 years, range 75 to 94 years).

### Interview analysis

Women appraised their breast cancer and potential treatment options (primary appraisals), albeit often only partially. They also considered and employed various coping mechanisms (secondary appraisals). Many did not perceive that they had a choice to make, either because they were not offered a choice by their healthcare professional, or they felt the decision was almost instantly obvious. The majority did not describe experiencing difficulty with decision making. Most formed a preference relatively quickly based on their past experiences and personal goals and values. Women’s appraisal and coping processes are described in more detail in the following sections and references are given to the appropriate CODE framework question adaptation with example quotes (refer to Tables 1, 2 and 3). Quotes are identified by the participant’s age and type of treatment (PET or surgery).

#### Breast cancer

##### Primary appraisals: threat or relevance of breast cancer (*Table**1**. A*)

Women’s cognitive appraisals of breast cancer were mainly focussed on their chances of survival and the potential for cancer control or cure. They often drew on their family members’ and friends’ experiences of cancer.

*“…I shut down regarding the breast cancer because I thought that was going to kill me…”*_77, PET_

Some explored the potential causes of their cancer and the impact of their diagnosis on their future plans.

Emotional appraisals of their breast cancer included shock and distress at the diagnosis for some, but also an acceptance of it, with women often remarking that getting the diagnosis later in life was not as bad as being diagnosed at a younger age.

*“I didn’t feel sorry for myself, I didn’t like it but I thought well at my age you’ve got to die sometime, I’ve gone a long time you know what I mean and I feel sorry for younger ones I do.”*_82, PET_

They also considered the impact it may have on their quality of life.

Interviewer: Were you upset? “*Yes you know, I mean it’s not a pleasant … I only was worrying about the pain I’m going to get in time…”*_90, PET_

##### Secondary appraisals: resources to cope with breast cancer (*Table* *1**. B*)

Many women did not wish to have a detailed understanding of their breast cancer and did not seek information in addition to that provided by their breast specialists. Some women described distracting themselves from their diagnosis; blocking conversations and thoughts about breast cancer. However, one woman sought additional information and advice from relatives.

Women considered talking to relatives and friends about their breast cancer, with this providing a source of instrumental and/or emotional support (especially in terms of accompanying them to their appointments).

*“Because my sister came with me, because she had breast cancer, but she had it over twenty years ago.”*_75, surgery_

Others, however, did not wish to discuss or “*burden*” relatives and friends. Some considered emotion-focused strategies such as positive reappraisal and their faith to cope with their breast cancer.

*“I just keep praying and talking to God about it and telling him all about it and everybody keep*[s] *praying for me…”*_76, PET_

#### Treatment choice

##### Primary appraisals: threat or relevance of choice (*Table* *2**. A*)

Women initially appraised whether there was a choice at all. If they perceived a choice had been offered, they explored the scope of the choice (i.e. how many options, what the options were) and timeline of the decision. Some talked about the choice between types of surgery and did not recall the offer of PET.

*“…he did give me the option of what he could do, he could just take the lump out, he could remove the breast, there’s something else.”* Interviewer: Hormone treatment? *“I don’t remember that but I know he said if I had just the lump taken, oh and he could also rebuild it at the same time if I wished..*.” _79, surgery_

Feelings about having a choice were also discussed. While some described difficulty with decision making and therefore did not want so much choice, others felt pleased that they were offered a choice and could participate in decision making.

*“I think choice is, I think you like to feel that your body is your own, even (though) you’re having things done to it.”*_80, PET_

##### Secondary appraisals: resources to cope with choice (*Table* *2**. B*)

Some women discussed their treatment decision with their healthcare professional and/or relatives and friends. They often allowed those individuals to help them reach a decision. Some described canvassing opinions or seeking advice from others.

*“…I said to my son-in-law, ‘Honestly* [son-in-law’s name]*, give me your opinion. Now do I really need the operation, or…?’ I didn't say I would go with what he said…”*_81 PET_

Others talked about making a decision independently from their family and friends, appraising that they were sufficiently equipped and confident to complete the task.

Some women turned to their faith to help them cope with the choice.

*“I feel if you pray, if you have a faith and you pray and then to put… you leave it in God’s hands.”*_98, PET_

#### Treatment options and preference construction

##### Primary appraisals: threat or relevance of treatment options (*Table* *3**. A*)

Some women did not appraise their treatment options in any detail, particularly those options which they rejected outright.

Appraisals that were made often included the risks and benefits of options, with a particular focus on chances of survival, side-effects and practical aspects.

*“…I think it’s very important that they give you the options and … for instance, to know how long do you think the tablets can keep the cancer away … and what’s, what’s the success rate is for being operated on, as well because that’s important in your choice.*_80, PET_

Women also appraised the potential negative impact of the options on their quality of life, which included their post-operative care, ability to cope with activities of daily living and ability to continue to enjoy their hobbies.

*“…I’m worried that my chief hobby is crown green bowling and about the lymph glands in my right arm…”*_79, PET_

Women tended not to be concerned about post-operative scarring and altered body image. However, some considered the impact of this and other physical changes, in particular hair thinning/loss. They often brought their age and fitness into their appraisals.

*“…I’ve had friends that have had surgery ... but they were younger. I didn’t think it was worth it at my age.”*_90, PET_

While appraising their options, most drew on past experiences of cancer (in some cases many years ago).

*“…what I’ve heard about it, people and friends going, I would not want to be going every day for radiotherapy somewhere. I thought this I cannot do.”*_90, PET_

When talking about surgery many reported feeling frightened and concerned about the surgical procedure and things associated with it (e.g. hospitals, doctors, anaesthetic). Also, some women specifically mentioned feelings about radiotherapy, chemotherapy and tablets.

*“Radiotherapy, I’d got a dread of that…”*_75, surgery_

##### Primary appraisals: preference construction (*Table* *3**. B*)

Preferences were formed mainly based on personal goals and values and/or doctor’s recommendation. Those who followed the doctor’s recommendation often chose to learn very little about the alternative option and emphasised the trust they put in their healthcare team. Of the women who played an active part in the decision, the goals of those who chose a mastectomy were to get rid of the cancer as soon and fully as possible as well as to avoid further treatment. The goals of those who chose PET were to avoid surgery (often due to their age or past experiences) and hospitalisation, as well as to preserve their current quality of life.

*“…I said, ‘I don’t want to live any longer, but I do want to stay in my own house as long as I possibly can’ …what I insisted on was, trying to give me the best quality of life they could give me…”*_85, PET_

While some women preferred PET, a couple also mentioned that it left their options open should PET be unsuccessful.

*“I’d say well if I had a choice I’d rather try a tablet first and then if nothing, if it wasn’t successful then I would have surgery.”*_8592, PET_

Many women formed their preferences very quickly, based on past experience and/or a gut feeling when diagnosed.

*“…straight away I just said ‘take it off’ and I meant take the lot off … they gave me a choice of treatments, and I said ‘just take it off, cut it out’.”*_84, surgery_

##### Secondary appraisals: resources to cope with treatment options and preference construction (*Table* *3**. C*)

While most women seemed to accept the information from healthcare professionals, some women sought additional information about the treatment options. Some also spoke to peers with first-hand experience of breast cancer and treatment.

*“…*[My friend who’d had surgery] *said come and see her you know so we both poled over and she bared all and she said she wouldn’t have a lumpectomy because of the radiotherapy which finished her off.”*_79, PET_

Some women considered whether they were strong or healthy enough to recover from surgery and explored whether there were additional coping strategies they could employ, such as dietary supplements or relaxation techniques. Women appraised instrumental coping resources that could help them post-surgery, for example whether family and friends would be able to provide practical support. Potential sources of emotional support were family, friends and religious beliefs.

## Discussion

Older women have previously been described as usually preferring a more passive role in treatment decision making [13]. However, the present study suggests that many were involved in their breast cancer treatment decision and used a number of strategies to support this process. Individual variation in degree of involvement ranged from women making the decision themselves, to making a joint decision with their specialist, to asking for or deferring to the specialist’s recommendation. The women interviewed did not appear to struggle with decision making about breast cancer treatment. Some made their decisions very quickly, some had pre-conceived ideas and others rapidly evaluated and rejected or accepted treatments offered, describing an immediate preference. Others deliberated to varying degrees, with differing levels of external support and discussion. Collaboration with healthcare professionals included receiving and seeking information, advice and discussion about the options and choice, joint decision making and help to cope with choosing.

Some women demonstrated active involvement in decision making, reporting strong feelings of knowing clearly what they wanted. In contrast, others followed the doctor’s treatment recommendation and these women often chose to learn very little about the options not recommended to them, suggesting trust in the healthcare team. However, rather than reflecting inactivity in decision making, the approach of this latter group of older women may allow them to conserve resources and use their experience to defer to the clinical expertise and knowledge of the healthcare professionals. The idea of deferring to clinical expertise is supported by Husain and colleagues [2] who reported that women sought guidance from their healthcare professionals. By including an examination of coping strategies when assessing the deliberation process, some decision making preferences previously judged as passive may be understood differently. As Aldwin [12] suggested, older adults may use more efficient coping strategies which can be very adaptive in later life. Some older women may therefore actively choose to use an effective coping strategy, such as deferring to the healthcare professional or consulting their social network, to make their decision. However, an alternative explanation may be that patient barriers, such as lack of confidence in asking questions, difficulty in understanding information and failure to understand that there is a choice [14], preclude active participation in treatment decision making.

Many made their decisions relatively quickly. Making fewer or faster appraisals is no less valid than taking a more deliberative and potentially longer, resource-intensive approach. Indeed, using limited information or *rule of thumb* approaches to decision making has been argued to be just as effective as more complex approaches [15–17].

Some women did not perceive that they had a choice to make because they were not offered a choice by their healthcare professional, yet the eligibility criteria included having been offered a choice of surgery or PET. There are at least three possible explanations for this. Firstly, an error may have occurred when reviewing the participant’s eligibility for the study. Secondly, the understanding of “offering a choice” might be understood differently among healthcare professionals. For example, for one it may mean that the patient was presented both options equally, asked about their preferences and the two options discussed, however for another it may mean that one treatment was recommended but an alternative was mentioned within the discussion. Thirdly, women may not recall the alternative being offered to them (and this might particularly be the case if they were not interested in the alternative) or may not have understood that they had a choice.

The CODE framework was found to be adaptable to breast cancer treatment decisions and enabled insights to be gained into older women’s decision making and coping. There were, however, limitations to the present study. This was a secondary analysis of data collected to assess older women’s information and decision support needs. The primary focus of the interviews was not on decision making and coping processes, hence full analysis of these processes was not possible. Biases may have been introduced from the limited data and interpretation by the coders. Retrospective recall bias is also possible as the eligibility criteria included diagnosis within the last five years, and some women reported a diagnosis prior to this, possibly demonstrating recall error. Finally, the treatment decisions discussed within the interviews were not always solely decisions between PET and surgery, but also included decisions between different types of surgery (with and without consideration of PET). Despite these limitations, the present findings provide a unique insight into older women’s appraisals about their breast cancer treatment decision and coping strategies, and the basis for a theoretically grounded DSI for these women.

An important implication of this study is that women should be offered decision support that recognises individual variation in decision making and coping, and supports them in achieving their preferred level of involvement. This might include raising questions such as “how much do I want to be involved in this decision?” within decision support material. Such an approach could potentially empower those who lack confidence to take up the opportunity to be involved and open up the option of seeking a recommendation from the healthcare professional for those who want it. Indeed, it might be important to recognise within a DSI that deferring to a healthcare professional’s recommendation is a valid decision making approach, and therefore effective coping strategy, if it is *preceded* by a discussion of the options available and of personal preferences [18]. As variation was found in decision quality in terms of lack of knowledge about the treatment options or indeed that a choice exists, a DSI which promotes shared decision making and clearly states the options may benefit women facing the choice of PET or surgery with endocrine therapy. Concise and easy to understand information relevant to the decision, such as an option grid [19] for use within the clinical setting, may help patients and healthcare professionals to engage in shared decision making. Information which is short and clear that can be used within the clinic setting may be particularly appropriate for women facing this decision who appear to make a decision relatively quickly, may not want much additional information other than what the healthcare professional provides and appreciate the discussion with healthcare professionals [3]. Longer components of a DSI may also be developed to provide an additional resource for those patients who wish to explore the information and options in more detail, enabling them to consolidate and build on their knowledge, and facilitate discussion with healthcare professionals and relatives/friends (if that is their preference). The dual approach of a concise and expanded DSI format may support fast, intuitive emotional responses as well as more deliberative, cognitive responses to information about treatment options [20].

Having adapted the CODE framework for the decision between PET and surgery with endocrine therapy, these questions can guide the content development of a DSI. For example, along with information on survival and recurrence rates, presenting the possible impact of each treatment option on quality of life may, for women facing the choice of PET or surgery with endocrine therapy, be important. Recognising the limitations of general health at a later stage in life and the effect of comorbid illnesses and medication may also need to be addressed. As past experiences were often reported by women when considering the treatment decision, highlighting that experiences might vary and that treatments may have changed over time would also be a useful addition to a DSI. Different coping strategies which have been identified through the adapted CODE framework can be encouraged and supported within a DSI. This may include suggestions to discuss the decision with others, or to seek strength or comfort in religious/spiritual beliefs.

Further research is needed to assess older women’s preferences for breast cancer treatment decision making within a larger sample. Collecting quantitative data would allow further generalisations to be made about involvement and preferences in breast cancer treatment decisions. It is also important to gain an understanding of healthcare professionals’ attitudes towards treatment decisions in order to be able to encourage shared decision making.

## Conclusions

Many older women with breast cancer are involved with their breast cancer treatment decisions and may benefit from DSIs designed to support collaborative decision making and coping, within and beyond the clinic setting. Using the CODE framework, appraisals of coping and decision making about breast cancer treatment were identified and key questions which older women may ask during the deliberation process were outlined. These will be informative for the development of DSIs to support older women making breast cancer treatment decisions.

## Abbreviations

CODE: Coping in Deliberation
DSI: Decision support intervention
PET: Primary Endocrine Therapy
